# Age differences in the association of physical leisure activities with incident disability among community-dwelling older adults

**DOI:** 10.1265/ehpm.21-00018

**Published:** 2022-03-31

**Authors:** Kimiko Tomioka, Midori Shima, Keigo Saeki

**Affiliations:** Nara Prefectural Health Research Center, Nara Medical University, Kashihara, Nara, Japan

**Keywords:** Incident disability, Leisure activities, Prospective study, Community-dwelling older adults

## Abstract

**Background:**

The relationship between leisure activities (LA) in old age and prevention of disability has not been fully investigated, and age and gender differences of these relationships are unknown. This study aimed to investigate whether physical and cognitive LA predicted incident disability among community-dwelling older adults by age and gender.

**Methods:**

We prospectively observed 8,275 residents aged 65 or above without disability at baseline for 3 years. Incident disability was defined as a new certification of the public long-term care insurance system. LA were classified into two types: physical LA and cognitive LA. The frequency of LA was categorized into frequent (i.e., once a week or more), moderate (i.e., monthly or yearly), and non-engagement. Covariates included age, gender, family number, education, perceived economic situation, body mass index, chronic medical conditions, alcohol consumption, smoking status, regular dental visits, depression, cognitive functioning, and social participation. Multivariable Poisson regression models were used to estimate adjusted cumulative incidence ratio (CIR) and 95% confidence interval (CI) for incident disability. We performed stratified analyses by age groups (i.e., the young-old aged 65–74 and the old-old aged 75–97) and gender (i.e., men and women).

**Results:**

The 3-year cumulative incidence of disability was 7.5%. After adjustment for covariates and mutual adjustment for both types of LA, a significant dose-response relationship between more frequent LA and lower risk of incident disability was found in young-old physical LA (*P*-trend < 0.001), in old-old cognitive LA (*P*-trend = 0.012), in male cognitive LA (*P*-trend = 0.006), and in female physical LA (*P*-trend = 0.030). Compared with people without LA, adjusted CIR (95% CI) of frequent LA was 0.47 (0.30–0.74) in young-old physical, 0.75 (0.58–0.96) in old-old cognitive, 0.65 (0.46–0.89) in male cognitive, and 0.70 (0.52–0.95) in female physical. Regarding the effect modification according to age and gender, only interaction between age and physical LA significantly prevented incident disability (*P* for interaction = 0.019).

**Conclusion:**

We found age differences in the association of physical LA with incident disability among community-dwelling older adults. An effective measure to prevent long-term care in the community would be to recommend frequent physical LA for the young-old.

**Supplementary information:**

The online version contains supplementary material available at https://doi.org/10.1265/ehpm.21-00018.

## Introduction

Healthy life expectancy is the number of years a person may expect to live independently without any health problems, and is a concept introduced by the World Health Organization [1]. Loss of healthy life expectancy, that is, having a disability, not only reduces the quality of life of the person, but also increases the burden on the family members who care for them. It also puts pressure on national finances due to the increase in social security costs such as medical expenses and nursing-care expenses. Therefore, prevention of disability is an urgent issue for countries with a high aging rate, including Japan. Research is needed to identify measures to prevent disability, which address lifestyle changes within community-dwelling older people.

In an aging society, being active is regarded as the most important measure to prevent disability [2], and many studies have reported that leisure activities (LA) in old age are associated with maintenance of cognitive function [3, 4]. A few studies have suggested that LA by older adults are associated with a lower risk of frailty [5, 6]. Because frailty has been associated with an increase in incident disability [7], LA may be a modifiable factor that prevents the onset of disability among older people. Although LA enjoyed by community-dwelling older adults differ depending on their age and gender [8, 9], age and gender differences in the association between LA and incident disability are unclear. Recently, researchers have developed questionnaires to evaluate the LA of older adults, through the classification of LA into physical, cognitive, and social components [9, 10]. Previous studies have reported that social participation, including the social component of LA, is a powerful preventive factor in the development of disability [11, 12]. Therefore, it is necessary to consider whether physical and cognitive LA prevent incident disability, independently of social participation.

In this study, using data from a community-based prospective cohort study, we examined whether physical and cognitive LA are predictors of incident disability among community-dwelling older adults, independently of social participation, by age and gender.

## Methods

### Study participants

**Fig. 1:**
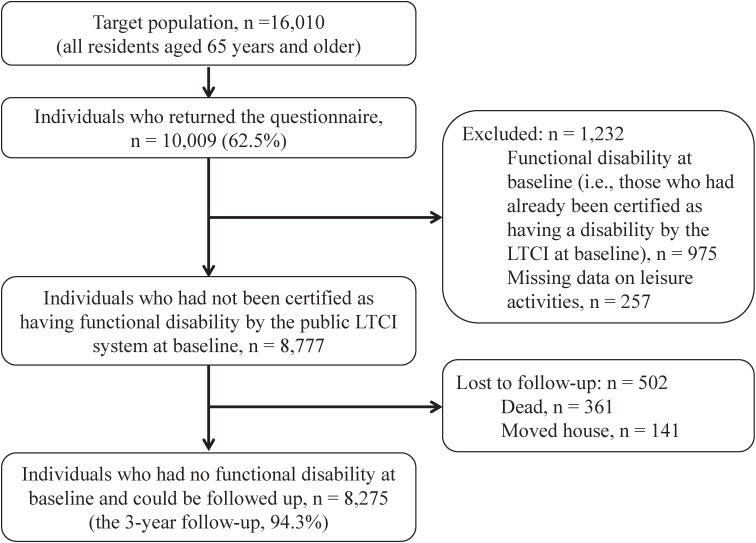
Flow chart of study participants. LTCI, Long-term Care Insurance

The details of this cohort study have been explained elsewhere [13, 14]. Briefly, potential participants of this study were all residents (n = 16,010) who were at least 65 years of age as of April 1, 2016, living in Municipality A in Nara Prefecture with a lower aging rate than the national average. A baseline questionnaire survey was conducted by mail in October 2016, and responses were obtained from 10,009 people (62.2%). A 3-year follow-up was conducted on 8,777 participants, excluding those who had already been certified as having a disability by the public long-term care insurance at the baseline survey (n = 975) and those who had a missing value in LA (n = 257). The final number of participants analyzed was 8,275, excluding those who died (n = 361) and those who moved house (n = 141) (Fig. 1). The 3-year follow-up rate was 94.3%.

First, we compared the basic attributes of those who responded to the baseline survey and those who did not (Additional file 1). Compared to the respondents, non-respondents were more likely to be in the youngest age group (65–69 years old), the oldest age group (85 years old or older), and those with functional disability. There was no gender difference in the presence or absence of response. Next, we compared the baseline characteristics of those who were or were not lost to follow up (Additional file 2). Compared to those with follow-up, participants lost to follow-up tended to be male, older, less educated, depressed, cognitively impaired, and unemployed.

### Assessment of incident disability

Incident disability was evaluated based on the certification status of the public long-term care insurance system. The public long-term care insurance system was established in 2000 with the aim of supporting long-term care in society as a whole, and its implementation lies with the municipalities [15]. When long-term care is needed, it is necessary to obtain long-term care certification in order to use the long-term care service. Certification for long-term care is based on the results of the certification assessment conducted by the investigator and the written opinion of the attending physician [15]. In the certification assessment process, interviews are conducted regarding the person’s physical and mental condition, and the content of the assessment is uniform throughout the country. Regarding the doctor's opinion, the attending physician prepares a written opinion on the person’s physical and mental condition. A prior study has confirmed that the long-term care certification has a strong correlation with the Barthel Index, which is frequently used as an evaluation index for basic activities of daily living [16]. Moreover, previous studies have reported that the new certification for long-term care is an effective index for the occurrence of disabilities among Japanese community-dwelling older adults [17, 18]. In this way, the evaluation of disability using the long-term care certification of the public long-term care insurance system is an objective and valid index.

In this study, individuals who had not been certified as having a disability by the public long-term care insurance at baseline were followed for 3 years, and those who were certified as requiring long-term care at the time of follow-up were judged to have incident disability.

### Assessment of physical and cognitive leisure activities (LA)

According to the Survey on Time Use and LA conducted by the Statistics Bureau of Japan [19], LA are defined as “activities carried out in one’s free time” and do not include activities that are physiologically necessary, such as sleeping and eating, and activities with strong obligatory elements in conducting social life such as paid work and housework. Therefore, in this study, LA were defined as activities carried out in one’s free time, and do not include activities that are necessary for daily living.

In our previous study, which used the same baseline data as the current study [13], we examined the cross-sectional association between the presence or absence of 14 types of LA and self-rated health. Eight of these 14 LA were based on LA used in the study by Takeda et al. [20]: physical activities, cultural activities, music activities, creative activities, gardening, TV watching, sightseeing, and investment/gambling. Additionally, referring to other studies on LA of older people [3, 21–23], we added four types of LA: art appreciation, playing games, cooking, and pet ownership. Furthermore, in recent years, the spread of information and communications technology has created a new lifestyle based on the Internet, e-mail, and mobile/smart phones. And with the recent reforms of the pension system and the impact of low interest rates, investment by older adults has become a common and important tool for asset management. Considering these recent changes in the living environment of older people, technology usage was added as one type of LA, and investment and gambling were classified as separate types of LA.

In this study, we used 5 of these 14 types of leisure activities: physical activities (walking, jogging, swimming, golf, ground golf, gate ball, tennis, gymnastics, dancing, and yoga), musical activities (playing a musical instrument, singing in a choir, folk singing, and karaoke singing), creative activities (handicraft, ceramics, painting, photography, and home carpentry), cultural activities (reading, learning, writing poems, flower arranging, tea ceremony, and calligraphy), and playing games (Go, Japanese chess, and mahjongg). Moreover, musical activities, creative activities, cultural activities, and playing games are classified as cognitive LA, because these activities have been reported to be associated with the prevention of cognitive decline. Indeed, some researchers have defined them as cognitive LA [3, 4, 21]. For the purposes of analysis, therefore, LA were classified into two types: physical LA and cognitive LA.

Respondents were asked about the presence or absence of activities and the frequency of activities for each of physical LA and cognitive LA. The frequency of LA consisted of the following five categories: almost daily, at least once a week, several times a month, and several times a year. Based on previous studies which classified activities carried out once a week or more as “frequent engagement” [17, 24], we classified LA into three categories: frequent = once a week or more, moderate = monthly or yearly, and non-engagement.

### Covariates

With reference to previous studies [3–5, 8–11, 21, 25], age, gender, socio-economic status (i.e., family number, education, and perceived economic situation), health status (i.e., body mass index and chronic medical conditions), health behaviors (i.e., alcohol consumption, smoking status, and regular dental visits), mental functioning (i.e., depression and cognitive functioning), and social participation (i.e., community activities, paid work, and informal social interactions) [11] were selected as covariates that may be confounding factors of the association between LA and incident disability. A representative survey of non-institutionalized individuals aged 60 and over in Spain reported that perceived economic situation was significantly associated with the prevalence of disability [26]. We therefore adopted perceived economic situation as an indicator of the economic affluence of socio-economic status.

Age was classified into 65–69, 70–74, 75–79, 80–84, or ≥85 years. Gender was dichotomized into male and female. Family number was categorized as one (i.e., living alone), 2, or ≥3 persons. Education (years of schooling) was categorized as ≤9, 10–12, or ≥13 years. Perceived economic situation was categorized as rich, middle, or poor. Body mass index (BMI) was categorized as underweight (<18.5), normal (18.5–24.9), or overweight (≥25.0). Chronic medical conditions included hypertension, stroke, heart disease, and diabetes. The number of chronic medical conditions under medical treatment was categorized as none, one, or ≥2. Alcohol consumption was classified with or without daily drinking habits. Smoking status was categorized as never-smokers, ex-smokers, or current smokers. Regular dental visits were defined as having visited the dentist for treatment and/or routine checkup at least once in the past year [25], and categorized as present or absent. Depression was assessed by the 5-item short form of the Geriatric Depression Scale [27], and categorized as present (scores of 2–5) or absent (scores of 0–1). Cognitive functioning was assessed by the MDS Cognitive Performance Scale [28], and categorized as poor (scores of 1–6) or intact (a score of 0). Community activities included volunteer activities and neighborhood association activities, and were classified with or without participation. Paid work was dichotomized into present (i.e., working) or absent (i.e., non-working). Informal social interactions were defined as the frequency of meeting friends and acquaintances, and categorized into more than once a month or less than once a month.

Regarding the variables used for covariates, we confirmed that there were no variables with a variance inflation factor greater than 5.0 and that there was no problem with multicollinearity.

### Multiple imputation

In this study, 1,061 people (12.8% of the participants analyzed) lacked at least one covariate data. Therefore, for missing covariate data, we performed multiple imputation by chained equations in order to reduce the possibility of inferential bias [29].

Using logistic regression, we created five sets of imputation data and performed analyses on the complete pooled data set. Age, gender, family number, education, perceived economic situation, BMI, chronic medical conditions, alcohol consumption, smoking status, regular dental visits, depression, cognitive functioning, community activities, paid work, informal social interactions, the independent variables (i.e., physical LA and cognitive LA), and outcome (i.e., incident disability) were entered into the imputation procedure. Multiple imputation was conducted using the IBM SPSS Missing Values Option.

### Statistical analyses

The Chi-squared test or the *t* test was used to examine the difference in categorical or continuous variables of baseline characteristics between the two groups.

The generalized estimating equations of the multivariable Poisson regression models were applied to estimate the cumulative incidence ratio (CIR) and 95% confidence interval (CI) for incident disability. The independent variables were physical LA and cognitive LA at baseline. First, we calculated a crude CIR for incident disability. Next, in Model 1, both types of LA and social participation were adjusted. Subsequently, in Model 2, gender, age, socio-economic status, health status, and health behaviors were added simultaneously. In the final Model 3, mental functioning was added to the variables in Model 2. The analyses were first performed on all the study participants and the three sets of models were used. Next, we performed stratified analyses by age groups (i.e., the young-old aged 65–74 and the old-old aged 75–97) and gender (i.e., men and women) using only the final Model 3.

Our results may be influenced by the correction of missing values using the multiple imputation method. Furthermore, the association between LA and incident disability can be affected by depression or working status. For example, older people with depressive symptoms participate in significantly fewer LA than those without depressive symptoms [30], and depressive symptoms may be a predictor of disability in the older population [31]. A meta-analysis reported that retired people had a stronger relationship between leisure engagement and subjective well-being compared with working people [32]. In order to examine the robustness of our findings, we conducted sensitivity analyses limited to participants without missing covariates, depression, or paid work.

Although in this study, we have focused on the association of the frequency of physical LA and cognitive LA with incident disability, and examined age and gender differences in these relationships, it is necessary to show results consistent with our previous study [13]. Therefore, we conducted additional analyses on the association between the presence or absence of 14 types of LA and incident disability and presented the analyzed results in an additional file (see Additional file 3).

Statistical analyses were conducted using the IBM SPSS Statistics Ver. 27 for Windows (Armonk, New York), and the level of significance was set at 0.05 (two-tailed test).

### Ethical Issues

This study was approved by the Nara Medical University Ethics Committee (approval number 939). Submission of self-completed questionnaires was considered agreement to participate in the research.

## Results

After the 3-year follow-up, there were 623 cases of incident disability, or the 3-year cumulative incidence of disability was 7.5%. The 3-year cumulative incidence of disability in the old-old (15.8%) was more than six times higher than that in the young-old (2.6%, *P* < 0.001). The 3-year cumulative incidence of disability was 6.7% in men and 8.3% in women, showing a significant gender difference (*P* = 0.007). Compared to people without incident disability, people with incident disability tended to live alone, have less years of education, have less normal weight, have more chronic illnesses, drink less daily, have less regular dental visits, have poorer mental functioning, have less social participation, and participate less in LA. There were no significant differences between the two groups in terms of subjective economic status and smoking habits (Table 1).

**Table 1:** Baseline characteristics by the incident disability during 3 years

	**Total** **(n = 8,275)**	**Incident disability**		***P* ^a^**

		**Absent** **(n = 7,652)**	**Present** **(n = 623)**	
Age, years, mean (SD)	73.3 (5.9)	72.7 (5.5)	79.8 (6.5)	<0.001
Gender: men (%)	46.2%	46.6%	40.9%	0.007
Socio-economic status^b^ (%)				
Family number: one	11.8%	11.2%	19.1%	<0.001
Education (years): ≤9	22.8%	22.0%	32.1%	<0.001
Perceived economic situation: poor	19.2%	19.3%	18.6%	0.728
Health status^b^ (%)				
Body mass index: 18.5–24.9	72.0%	72.5%	65.5%	<0.001
Chronic diseases^c^: present	62.0%	61.4%	68.9%	<0.001
Health behaviors^b^ (%)				
Alcohol: daily drinkers	26.9%	27.3%	21.8%	0.003
Smoking: current smokers	9.3%	9.4%	8.2%	0.313
Regular dental visits: present	51.2%	51.9%	42.2%	<0.001
Mental functioning^b^ (%)				
Depression: present	23.5%	22.0%	41.1%	<0.001
Cognitive functioning: poor	15.8%	14.4%	33.9%	<0.001
Social participation^b^ (%)				
Participation in community activities	47.6%	48.5%	37.2%	<0.001
Participation in paid work	22.6%	23.7%	9.0%	<0.001
Informal social interactions^d^: rarely^e^	25.5%	24.9%	32.1%	<0.001
Leisure activities (%)				
Physical leisure activities: involved	61.2%	62.3%	47.5%	<0.001
Cognitive leisure activities: involved	55.9%	56.5%	48.6%	<0.001

*SD* standard deviation.

^a^Means and proportions between the two groups were compared using the *t* test and the Chi-squared test.

^b^Based on the pooled data by multiple imputations.

^c^Chronic diseases included hypertension, stroke, heart disease, and diabetes.

^d^Informal social interactions mean frequency of meeting friends and acquaintances.

^e^Less than once a month.

Regarding the association between the type of LA and 3-year incident disability in all participants, in the crude model, people with more frequent LA were less likely to have incident disability than people without LA in both physical LA and cognitive LA. After adjustment for the covariates, the associations remained significant. (Table 2). For sensitivity analyses, which were limited to participants without missing covariates, depression, or paid work, similar results were observed (Additional file 4).

**Table 2:** CIR of LA’ types for 3-year incident disability in all the study participants

**Types of LA**	**n**	**Cumulative incidence^a^**	**Crude**	**Model 1**	**Model 2**	**Model 3**
			**CIR (95% CI)**	**CIR^b^ (95% CI)**	**CIR^c^ (95% CI)**	**CIR^d^ (95% CI)**
Physical leisure activities						
Without	3,208	10.2%	1.00	1.00	1.00	1.00
Moderate^e^	2,425	7.7%	0.76 (0.64–0.90)^*^	0.81 (0.69–0.97)^*^	0.98 (0.83–1.16)	0.99 (0.84–1.17)
Frequent^f^	2,642	4.1%	0.40 (0.33–0.50)^*^	0.45 (0.36–0.56)^*^	0.73 (0.58–0.91)^*^	0.76 (0.61–0.96)^*^
*P* for trend			<0.001	<0.001	0.010	0.032
Cognitive leisure activities						
Without	3,648	8.8%	1.00	1.00	1.00	1.00
Moderate^e^	2,637	7.7%	0.87 (0.74–1.03)	0.95 (0.80–1.12)	0.85 (0.72–1.00)^†^	0.86 (0.73–1.01)^†^
Frequent^f^	1,990	5.1%	0.58 (0.47–0.72)^*^	0.70 (0.56–0.87)^*^	0.71 (0.57–0.88)^*^	0.76 (0.61–0.95)^*^
*P* for trend			<0.001	0.002	0.001	0.010

CI, confidence interval; CIR, cumulative incidence ratio. ^*^P < 0.05. ^†^*P* < 0.10.

^a^The cumulative incidence of incident disability during the 3-year follow-up.

^b^Model 1 is adjusted for both types of leisure activities and social participation (i.e., community activities, paid work, and informal social interactions).

^c^Model 2 is adjusted for the variables in Model 1 plus gender, age (5-year increase), socio-economic status (i.e., family number, education, and perceived economic situation), health status (i.e., body mass index and chronic medical conditions), and health behaviors (i.e., alcohol consumption, smoking status, and regular dental visits).

^d^Model 3 is adjusted for the variables in Model 2 plus mental functioning (i.e., depression and cognitive functioning).

^e^Monthly or yearly. ^f^Weekly or more.

Regarding crude CIR for incident disability stratified by age and gender, more frequent LA was significantly related to a lower risk of incident disability, regardless of types of LA, age, and gender; *P* for trend was 0.026 in young-old cognitive LA, 0.007 in male cognitive LA, and <0.001 in all but these two (Tables 3 and 4).

**Table 3:** Stratified analyses by age using the crude and fully-adjusted models

**Types of LA**	**The young-old aged 65 to 74 years (n = 5,199)**				**The old-old aged 75 to 97 years (n = 3,076)**			
	
	**n**	**Cumulative incidence^a^**	**Crude CIR** **(95% CI)**	**Adjusted CIR^b^** **(95% CI)**	**n**	**Cumulative incidence^a^**	**Crude CIR** **(95% CI)**	**Adjusted CIR^b^** **(95% CI)**
Physical leisure activities								
Without	1,865	4.0%	1.00	1.00	1,343	18.8%	1.00	1.00
Moderate^c^	1,493	2.2%	0.55 (0.37–0.82)^*^	0.60 (0.40–0.90)^*^	932	16.5%	0.88 (0.73–1.06)	1.13 (0.94–1.36)
Frequent^d^	1,841	1.6%	0.39 (0.26–0.60)^*^	0.47 (0.30–0.74)^*^	801	10.0%	0.53 (0.42–0.67)^*^	0.90 (0.70–1.16)
*P* for trend			<0.001	<0.001			<0.001	0.721
Cognitive leisure activities								
Without	2,363	3.1%	1.00	1.00	1,285	19.1%	1.00	1.00
Moderate^c^	1,533	2.5%	0.79 (0.54–1.16)	0.87 (0.59–1.28)	1,104	14.9%	0.78 (0.65–0.93)^*^	0.84 (0.70–1.00)^†^
Frequent^d^	1,303	1.9%	0.61 (0.39–0.96)^*^	0.80 (0.49–1.29)	687	11.1%	0.58 (0.46–0.74)^*^	0.75 (0.58–0.96)^*^
*P* for trend			0.026	0.324			<0.001	0.012

CI, confidence interval; CIR, cumulative incidence ratio. ^*^P < 0.05. ^†^*P* < 0.10.

^a^The cumulative incidence of incident disability during the 3-year follow-up.

^b^Adjusted for covariates (i.e., gender, age, socio-economic status, health status, health behaviors, mental functioning, and social participation) and both types of leisure activities.

^c^Monthly or yearly. ^d^Weekly or more.

**Table 4:** Stratified analyses by gender using the crude and fully-adjusted models

**Types of LA**	**Men (n = 3,821)**				**Women (n = 4,454)**			
	
	**n**	**Cumulative incidence^a^**	**Crude CIR** **(95% CI)**	**Adjusted CIR^b^** **(95% CI)**	**n**	**Cumulative incidence^a^**	**Crude CIR** **(95% CI)**	**Adjusted CIR^b^** **(95% CI)**
Physical leisure activities								
Without	1,268	8.3%	1.00	1.00	1,940	11.4%	1.00	1.00
Moderate^c^	1,260	7.5%	0.90 (0.69–1.18)	1.05 (0.81–1.36)	1,165	8.0%	0.70 (0.55–0.88)^*^	0.95 (0.76–1.18)
Frequent^d^	1,293	4.3%	0.52 (0.38–0.72)^*^	0.84 (0.60–1.17)	1,349	3.9%	0.34 (0.26–0.46)^*^	0.70 (0.52–0.95)^*^
*P* for trend			<0.001	0.362			<0.001	0.030
Cognitive leisure activities								
Without	1,840	7.4%	1.00	1.00	1,808	10.1%	1.00	1.00
Moderate^c^	980	7.2%	0.97 (0.74–1.28)	0.79 (0.61–1.03)^†^	1,657	7.9%	0.78 (0.63–0.97)^*^	0.91 (0.74–1.12)
Frequent^d^	1,001	4.7%	0.63 (0.46–0.87)^*^	0.65 (0.46–0.89)^*^	989	5.5%	0.54 (0.40–0.72)^*^	0.88 (0.66–1.18)
*P* for trend			0.007	0.006			<0.001	0.335

CI, confidence interval; CIR, cumulative incidence ratio. ^*^P < 0.05. ^†^*P* < 0.10.

^a^The cumulative incidence of incident disability during the 3-year follow-up.

^b^Adjusted for covariates (i.e., age, socio-economic status, health status, health behaviors, mental functioning, and social participation) and both types of leisure activities.

^c^Monthly or yearly. ^d^Weekly or more.

According to the stratified analyses by age groups using the fully-adjusted model, among the young-old aged 65–74, a significant association between more frequent LA and a lower risk of incident disability was found only in physical LA, but not in cognitive LA. The CIRs (95% CIs) of moderate physical LA and frequent physical LA were 0.60 (0.40–0.90) and 0.47 (0.30–0.74), respectively, compared to people without physical LA, showing a significant dose-response relationship (*P* for trend <0.001). In contrast, among the old-old aged 75 and older, an inverse association between frequent LA and incident disability was observed only in cognitive LA, but not in physical LA. The CIRs (95% CIs) of moderate cognitive LA and frequent cognitive LA were 0.84 (0.70–1.00) and 0.75 (0.58–0.96), respectively, compared to people without cognitive LA, showing a significant dose-response relationship (*P* for trend = 0.012) (Table 3).

According to the stratified analyses by gender using the fully-adjusted model, among men, a significant association between more frequent LA and a lower risk of incident disability was observed only in cognitive LA, but not in physical LA. The CIRs (95% CIs) of moderate cognitive LA and frequent cognitive LA were 0.79 (0.61–1.03) and 0.65 (0.46–0.89), respectively, compared to people without cognitive LA, showing a significant dose-response relationship (*P* for trend = 0.006). In contrast, among women, an inverse association between frequent LA and incident disability was found only in physical LA, but not in cognitive LA. The CIRs (95% CIs) of moderate physical LA and frequent physical LA were 0.95 (0.76–1.18) and 0.70 (0.52–0.95), respectively, compared to people without physical LA, showing a significant dose-response relationship (*P* for trend = 0.030) (Table 4).

Regarding the interaction between age and each type of LA, an interaction effect by age was significant in physical LA (*P* for interaction = 0.019), but non-significant in cognitive LA (*P* for interaction = 0.767). Regarding the interaction between gender and each type of LA, neither physical LA nor cognitive LA had an interaction effect by gender (*P* for interaction was 0.388 in physical LA and 0.351 in cognitive LA). These results showed that the association of physical LA with incident disability was different by age but was not statistically different by gender, and that the association of cognitive LA with incident disability was not statistically different by age and gender.

## Discussion

This study examined the association of physical and cognitive LA with incident disability, using a community-based prospective cohort study of older adults. First, among all the study participants, a significant association between more frequent LA and lower risk of incident disability was observed in both physical and cognitive LA. Second, after stratified analyses by age and gender, a significant dose-response relationship was found in physical LA among the young-old, in cognitive LA among the old-old, in cognitive LA among men, and in physical LA among women. Third, the examinations of the interaction terms indicated that the young-old were more likely than the old-old to avoid incident disability by engaging in physical LA. These results were independent of social participation and each type of LA, which are known to be preventive factors for disability onset [11, 12, 20, 24]. To our knowledge, this is the first study to demonstrate age differences in the association of physical LA with incident disability among community-dwelling older adults.

Regarding comparison with previous research, a prospective cohort study of older people aged 70 and over recruited nonrandomly from communities reported that the combination of physical activity and cognitive activity reduced the risk of disability onset, but engaging in either physical activity or cognitive activity was not associated with incident disability [33]. Because this previous study [33] targeted active and healthy older people due to the convenience sample and had a short follow-up period of 2 years, it may lead to no single effect of physical activity on disability onset. In addition, this study did not perform a stratified analysis by age.

Many cohort studies of community-dwelling older adults have reported that physical LA can prevent the development of incident disability [2, 34], but age and gender differences have not been investigated. Two studies of people under the age of 65 have suggested that physical LA may prevent disability onset in men. First, one study which followed community-dwelling adults aged 64–65 at baseline for about 12 years found that men with a high level of leisure-time physical activity had a lower risk of disability onset with dementia compared to the no activity group, while women had no association between leisure-time physical activity and disability onset [35]. Second, an ecological study in Japan found that there was a positive correlation between middle-aged people’s involvement in exercise or sports and healthy life expectancy at the prefecture level, and that this relationship was stronger for men than for women [36]. We suggest three reasons why the results of gender-specific analyses in previous studies do not match the results of this study (i.e., why there was no significant gender difference in the association between physical LA and incident disability). First, there may be differences in outcome indicators, the age of study participants, study design, and follow-up duration. Second, because older people often lose their social role and have more time to spend on LA than middle-age people, LA in old age may have a different effect on disability than LA in middle age [13]. Third, middle-aged women spend more time in domestic work such as housework, childcare, and nursing than middle-aged men, which limits the amount of time they can devote to exercise or sports, leading to a weaker association between physical LA and disability in women than in men [35].

Although the mechanisms underlying age differences in the association of physical LA with incident disability are not fully understood, we have three possible explanations. First, physical activity in old age can reduce the risk of incident disability [2, 34], but this effect is only applied in higher-intensity physical activity [37]. Previous studies have reported that the proportion of those engaging in moderate- to vigorous-intensity physical activity is significantly higher in the young-old than in the old-old [38, 39]. Therefore, the association between frequent physical LA and lower risk of developing disability may be found in the young-old, but not in the old-old. Second, physical LA not only has a beneficial effect on the health of older adults, but also has adverse effects such as increasing the risk of injuries and falls [40]. Because harmful physical effects due to physical LA occur commonly with aging [37], the old-old may receive less benefit from physical LA than the young-old, resulting in a significant interaction effect by age in physical LA. Third, the Comprehensive Survey of Living Conditions, which is a large nationally representative survey in Japan, asks people in need of long-term care the reasons why they need long-term care. According to the latest 2019 survey [41], regarding the main causes of need for long-term care by age, cerebrovascular disease ranks first among the young-old. Risk factors that cause cerebrovascular disease include hypertension, diabetes, and obesity [42]. Moderate aerobic exercise produces prophylactic and ameliorating effects on these risk factors, resulting in prevention of cerebrovascular disease [43]. Therefore, cerebrovascular disease, which is the main cause of the need for long-term care in the young-old, may be prevented by the frequent physical LA of the young-old, leading to a greater effect of physical LA on prevention of incident disability in the young-old than in the old-old.

This study has several strengths. First, it is a prospective cohort study of all older people living in a community. Second, it has a high follow-up rate of 94.3%. Third, this study has succeeded in controlling for important confounding factors in the association between LA and incident disability. The former two guarantee the generalization of the results of this study. The third emphasizes an independent association between LA and incident disability among non-disabled community-dwelling older adults.

On the other hand, this study has some limitations. First, the response rate at the baseline survey was 62.5%, which is insufficiently high. Compared to the respondents, the non-respondents were more likely to be 65–69 years old and 85 years old or older (data are presented in Additional file 1). In other words, this study’s participants selectively lacked the low-risk group and the high-risk group for incident disability and inactive LA among the community-dwelling older adults. Therefore, it is unclear that the results of this study are underestimated or overestimated. Second, the explanatory variables and covariates of this study were obtained from the self-administered questionnaires. The effects of bias due to self-reporting may have led our results towards a null association. Third, since LA were evaluated only at baseline, this study did not take into account changes in LA. Future research needs to investigate the relationship between changes in LA and incident disability. Finally, the 3-year follow-up period in this study may not have been long enough to examine age and gender differences in the association between LA and incident disability. In the future, studies with longer follow-up periods are needed to confirm age and gender differences in the association of physical and cognitive LA with incident disability.

Our findings have important implications for policy-makers and general practitioners. Policy-makers may promote physical LA tailored to the age of community-dwelling older adults as a measure to postpone the need for long-term care. General practitioners may advise the young-old patients to actively engage in physical LA to prevent deterioration in functional abilities.

## Conclusions

Our study has revealed that more frequent LA are associated with a lower risk of incident disability, independently of social participation, and that engagement in physical LA is more effective in preventing incident disability in the young-old than in the old-old. These findings suggest that community-dwelling older people can reduce the risk of long-term care by frequent participation in LA, and that the young-old in particular can benefit greatly from frequent physical LA.

## Abbreviations

BMI: body mass index
CI: confidence interval
CIR: cumulative incidence ratio
LA: leisure activities

## Supplementary information

**Additional file 1:** Basic attributes of respondents and non-respondents to the questionnaire.

**Additional file 2:** Baseline characteristics of those who could be followed up and those who were lost to follow-up.

**Additional file 3:** Adjusted CIRs for incident disability associated with 14 types of LA.

**Additional file 4:** Sensitivity analyses: restricting analyses to participants without missing covariates, depression, or paid work.

## Declarations

### Availability of data and materials

Data cannot be shared for ethical reasons: we did not obtain informed consent from the participants for the open use of individual data.
