# Supplementary material for: Age differences in the association of physical leisure activities with incident disability among community-dwelling older adults
Source: Environ Health Prev Med. 2022 Mar 31;27:16. doi: 10.1265/ehpm.21-00018 (PMC9251618; doi:10.1265/ehpm.21-00018)
Supplement: Supplementary file 1 — Additional file 1: Basic attributes of respondents and non-respondents to the questionnaire. [file ehpm-27-016-s001.docx]

Additional file 1. Basic attributes of respondents and non-respondents to the questionnaire

| Basic attributes | | Respondents | Non-respondents | *P*-value^a^ |
| --- | --- | --- | --- | --- |
|  |  | (n = 10,006) | (n = 6,004) |  |
|  |  | n (%) | n (%) |  |
| Gender | |  |  |  |
|  | Men | 4,570 (45.7) | 2,695 (44.9) | 0.342 |
|  | Women | 5,436 (54.3) | 3,309 (55.1) |  |
| Age (years) | |  |  |  |
|  | 65–69 | 3,030 (30.3) | 1,984 (33.0) | <0.001 |
|  | 70–74 | 2,623 (26.2) | 1,412 (23.5) |  |
|  | 75–79 | 2,135 (21.3) | 1,117 (18.6) |  |
|  | 80–84 | 1,266 (12.7) | 772 (12.9) |  |
|  | 85+ | 952 (9.5) | 719 (12.0) |  |
| Functional disability at baseline survey^b^ | | | |  |
|  | Absent | 9,032 (90.3) | 5,084 (84.7) | <0.001 |
|  | Present | 974 (9.7) | 920 (15.3) |  |

^a^Chi-squared test.

^b^Functional disability was deﬁned as persons who had already been certified as having a disability by the long-term care insurance as of November 30, 2016.
