# Supplementary material for: Age differences in the association of physical leisure activities with incident disability among community-dwelling older adults
Source: Environ Health Prev Med. 2022 Mar 31;27:16. doi: 10.1265/ehpm.21-00018 (PMC9251618; doi:10.1265/ehpm.21-00018)
Supplement: Supplementary file 2 — Additional file 2: Baseline characteristics of those who could be followed up and those who were lost to follow-up. [file ehpm-27-016-s002.docx]

Additional file 2. Baseline characteristics of those who could be followed up and those who were lost to follow-up

| Baseline characteristics | | Followed up | | Loss to follow-up^a^ | *P*-value^b^ |
| --- | --- | --- | --- | --- | --- |
|  |  | n (%) | | n (%) |  |
| Gender | |  | |  |  |
|  | Men | 3,821 (46.2) | | 395 (52.0) | 0.002 |
|  | Women | 4,454 (53.8) | | 364 (48.0) |  |
| Age (years) | |  | |  |  |
|  | 65–69 | 2,817 (34.0) | | 159 (20.9) | <0.001 |
|  | 70–74 | 2,382 (28.8) | | 147 (19.4) |  |
|  | 75–79 | 1,786 (21.6) | | 179 (23.6) |  |
|  | 80–84 | 870 (10.5) | | 155 (20.4) |  |
|  | 85+ | 420 (5.1) | | 119 (15.7) |  |
| Education (years) | |  | |  |  |
|  | ≥10 | 6,345 (77.3) | | 499 (67.9) | <0.001 |
|  | ≤9 | 1,867 (22.7) | | 236 (32.1) |  |
| Depression^c^ | |  | |  |  |
|  | Absent | 6,300 (76.5) | | 487 (65.4) | <0.001 |
|  | Present | 1,931 (23.5) | | 258 (34.6) |  |
| Cognitive functioning^c^ | | |  | |  |
|  | Intact | 6,784 (84.1) | | 490 (69.3) | <0.001 |
|  | Poor | 1,278 (15.9) | | 217 (30.7) |  |
| Paid work^c^ | |  | |  |  |
|  | Not involved | 6,334 (77.4) | | 605 (82.8) | <0.001 |
|  | Involved | 1,851 (22.6) | | 126 (17.2) |  |

Data are given as n (%).

^a^ Including individuals who were excluded because of missing data on leisure activities (n = 257) or being lost at follow-up (n = 502), but not including individuals who had functional disability at baseline (n = 975).

^b^Chi-squared test.

^c^Limited to individuals with valid response for this item.
