# Supplementary material for: Age differences in the association of physical leisure activities with incident disability among community-dwelling older adults
Source: Environ Health Prev Med. 2022 Mar 31;27:16. doi: 10.1265/ehpm.21-00018 (PMC9251618; doi:10.1265/ehpm.21-00018)
Supplement: Supplementary file 3 — Additional file 3: Adjusted CIRs for incident disability associated with 14 types of LA. [file ehpm-27-016-s003.docx]

Additional file 3. Adjusted CIRs for incident disability associated with 14 types of LA

|  |  |  | Stratified analyses by age | | | |  | Stratified analyses by gender | | | |
| --- | --- | --- | --- | --- | --- | --- | --- | --- | --- | --- | --- |
|  | All (n = 8,275) |  | Young-old (n = 5,199) |  | Old-old (n = 3,079) | P for  interaction |  | Men (n = 3,821) |  | Women (n = 4,454) | P for  interaction |
|  | Adjusted CIR^a^ |  | Adjusted CIR^a^ |  | Adjusted CIR^a^ |  |  | Adjusted CIR^b^ |  | Adjusted CIR^b^ |  |
| Sports activities | 0.95 (0.81-1.11) |  | 0.59 (0.41-0.84)^*^ |  | 1.10 (0.92-1.31) | 0.020 |  | 1.01 (0.79-1.30) |  | 0.88 (0.70-1.09) |  |
| Gardening | 0.87 (0.74-1.01)^†^ |  | 0.86 (0.61-1.23) |  | 0.86 (0.73-1.02)^†^ |  |  | 0.79 (0.61-1.02)^†^ |  | 0.95 (0.78-1.15) |  |
| Musical activities | 0.87 (0.72-1.04) |  | 0.73 (0.46-1.17) |  | 0.88 (0.72-1.07) |  |  | 1.01 (0.74-1.36) |  | 0.85 (0.68-1.06) |  |
| Creative activities | 0.74 (0.59-0.92)^*^ |  | 0.87 (0.52-1.43) |  | 0.72 (0.56-0.91)^*^ | 0.651 |  | 0.76 (0.50-1.16) |  | 0.75 (0.58-0.97)^*^ | 0.981 |
| Cultural activities | 1.15 (0.96-1.37) |  | 1.53 (1.04-2.26)^*^ |  | 1.03 (0.85-1.26) | 0.503 |  | 1.04 (0.77-1.40) |  | 1.19 (0.95-1.48) |  |
| Playing games | 1.00 (0.78-1.27) |  | 0.93 (0.53-1.66) |  | 1.01 (0.78-1.32) |  |  | 0.97 (0.73-1.28) |  | 1.05 (0.63-1.75) |  |
| Sightseeing | 0.92 (0.77-1.11) |  | 1.05 (0.72-1.55) |  | 0.87 (0.71-1.06) |  |  | 1.05 (0.80-1.37) |  | 0.82 (0.65-1.03)^†^ |  |
| Art appreciation | 0.94 (0.78-1.14) |  | 0.67 (0.44-1.03)^†^ |  | 1.06 (0.86-1.30) |  |  | 0.73 (0.52-1.01)^†^ |  | 1.13 (0.89-1.43) |  |
| TV watching | 1.02 (0.88-1.19) |  | 1.14 (0.79-1.64) |  | 1.00 (0.85-1.18) |  |  | 1.10 (0.86-1.42) |  | 0.98 (0.81-1.19) |  |
| Cooking | 1.13 (0.90-1.40) |  | 1.06 (0.65-1.73) |  | 1.14 (0.90-1.45) |  |  | 1.00 (0.58-1.73) |  | 1.11 (0.87-1.42) |  |
| Pet ownership | 1.12 (0.87-1.43) |  | 1.10 (0.70-1.73) |  | 1.13 (0.84-1.50) |  |  | 1.04 (0.70-1.55) |  | 1.18 (0.86-1.61) |  |
| Technology usage | 0.80 (0.63-1.03)^†^ |  | 0.94 (0.62-1.43) |  | 0.75 (0.55-1.02)^†^ |  |  | 0.83 (0.60-1.14) |  | 0.76 (0.52-1.13) |  |
| Investment | 0.96 (0.72-1.27) |  | 1.30 (0.77-2.21) |  | 0.87 (0.62-1.21) |  |  | 0.80 (0.54-1.18) |  | 1.18 (0.78-1.78) |  |
| Gambling | 0.75 (0.56-1.00)^*^ |  | 0.82 (0.46-1.46) |  | 0.69 (0.49-0.96)^*^ | 0.652 |  | 0.59 (0.40-0.86)^*^ |  | 1.19 (0.78-1.79) | 0.005 |

CI, confidence interval; LA, leisure activities; CIR, cumulative incidence ratio. ^*^ P <0.05. ^†^P <0.10.

^a^ Adjusted for covariates (i.e., gender, age, socio-economic status, health status, health behaviors, depression, cognitive functioning, and social participation) and engagement in all 14 types of LA.

^b^ Adjusted for covariates (i.e., age, socio-economic status, health status, health behaviors, depression, cognitive functioning, and social participation) and engagement in all 14 types of LA.
