# Supplementary material for: Age differences in the association of physical leisure activities with incident disability among community-dwelling older adults
Source: Environ Health Prev Med. 2022 Mar 31;27:16. doi: 10.1265/ehpm.21-00018 (PMC9251618; doi:10.1265/ehpm.21-00018)
Supplement: Supplementary file 4 — Additional file 4: Sensitivity analyses: restricting analyses to participants without missing covariates, depression, or paid work. [file ehpm-27-016-s004.docx]

Additional file 4. Sensitivity analyses: restricting analyses to participants without missing covariates, depression, or paid work

|  |  | Restricted to participants without | | |  | Restricted to participants without | | |  | Restricted to participants without | | |
| --- | --- | --- | --- | --- | --- | --- | --- | --- | --- | --- | --- | --- |
|  |  | missing covariate data (n = 7,214) | | |  | depression (n = 6,332) | | |  | paid work (n = 6,406) | | |
|  |  | n | Cumulative  incidence^a^ | CIR^b^ (95% CI) |  | n | Cumulative  incidence^a^ | CIR^c^ (95% CI) |  | n | Cumulative  incidence^a^ | CIR^d^ (95% CI) |
| Physical LA | |  |  |  |  |  |  |  |  |  |  |  |
|  | Without | 2,724 | 9.3% | 1.00 |  | 2,177 | 8.3% | 1.00 |  | 2,462 | 12.2% | 1.00 |
|  | Moderate^e^ | 2,112 | 6.8% | 0.92 (0.76–1.12) |  | 1,862 | 5.6% | 0.92 (0.73–1.15) |  | 1,841 | 8.9% | 0.98 (0.82-1.17) |
|  | Frequent^f^ | 2,378 | 3.7% | 0.71 (0.56–0.92)^*^ |  | 2,293 | 3.5% | 0.72 (0.55–0.95)^*^ |  | 2,103 | 4.8% | 0.80 (0.63-1.01) ^†^ |
|  | *P* for trend | |  | 0.010 |  |  |  | 0.021 |  |  |  | 0.077 |
| Cognitive LA | |  |  |  |  |  |  |  |  |  |  |  |
|  | Without | 3,142 | 7.9% | 1.00 |  | 2,560 | 6.7% | 1.00 |  | 2,743 | 10.6% | 1.00 |
|  | Moderate^e^ | 2,286 | 7.0% | 0.86 (0.71–1.03) |  | 2,061 | 5.6% | 0.74 (0.59–0.94)^*^ |  | 2,077 | 9.0% | 0.88 (0.74-1.04) |
|  | Frequent^f^ | 1,786 | 4.3% | 0.67 (0.52–0.87)^*^ |  | 1,711 | 4.7% | 0.78 (0.60–1.02)^†^ |  | 1,586 | 5.6% | 0.75 (0.60-0.95) ^*^ |
|  | *P* for trend | |  | 0.002 |  |  |  | 0.037 |  |  |  | 0.012 |

CI, confidence interval; LA, leisure activities; CIR, cumulative incidence ratio. ^*^ *P* <0.05. ^†^*P* <0.10.

^a^ The cumulative incidence of incident disability during the 3-year follow-up.

^b^ Adjusted for covariates (i.e., gender, age, socio-economic status, health status, health behaviors, depression, cognitive functioning, and social participation) and engagement in both types of LA.

^c^ Adjusted for covariates (i.e., gender, age, socio-economic status, health status, health behaviors, cognitive functioning, and social participation) and engagement in both types of LA.

^d^ Adjusted for covariates (i.e., gender, age, socio-economic status, health status, health behaviors, depression, cognitive functioning, and social participation excluding paid work) and engagement in both types of LA.

^e^ Monthly or yearly. ^f^ Weekly or more.
